# Salivary gland ultrasonography in primary Sjögren’s syndrome from diagnosis to clinical stratification: a multicentre study

**DOI:** 10.1186/s13075-021-02689-3

**Published:** 2021-12-20

**Authors:** Xia Zhang, Ruiling Feng, Jinxia Zhao, Yu Wang, Juan He, Li Liu, Yongjing Cheng, Haihong Yao, Sumei Tang, Jiali Chen, Shanshan Zhang, Zhiyi Zhang, Qingwen Wang, Jing He, Zhanguo Li

**Affiliations:** 1 Department of Rheumatology and Immunology, Peking University People’s Hospital, Beijing, China; 2 Department of Rheumatology and Immunology, Peking University Third Hospital, Beijing, China; 3 Department of Rheumatology and Immunology, Harbin Medical University First Hospital, Harbin, China; 4 Department of Rheumatology and Immunology, Peking University Shenzhen Hospital, Shenzhen, China; 5 Department of Ultrasound, Peking University Shenzhen Hospital, Shenzhen, China; 6 Department of Rheumatology and Immunology, Beijing Hospital, Beijing, China; 7 Department of Ultrasound, Peking University People’s Hospital, Beijing, China; 8 Peking-Tsinghua Center for Life Sciences, Beijing, China

**Keywords:** Sjögren’s syndrome, Salivary glands, Ultrasonography, Diagnosis

## Abstract

**Background:**

To determine the diagnostic accuracy of major salivary gland ultrasonography (SGUS) in primary Sjögren’s syndrome (pSS) using the novel Outcome Measures in Rheumatology Clinical Trials (OMERACT) scoring system in a large-scale multicentre study.

**Methods:**

SGUS was conducted for 246 pSS patients, 140 control subjects with conditions other than SS and 27 healthy control subjects. The echostructure features from the parotid and submandibular glands on both sides were graded using the novel OMERACT scoring system. Receiver operating characteristic curves were used to describe the diagnostic accuracy of the scoring system for pSS. The associations between the SGUS and disease characteristics were analysed to evaluate the clinical value of SGUS for pSS.

**Results:**

The US scores in the pSS group were significantly higher than those in the non-pSS group (*p* < 0.001). The level of diagnostic accuracy was comparable with the scores of all four glands (AUC=0.908) when only the parotid and submandibular glands on either side were scored (AUC=0.910, 0.904, respectively). The optimal cut-off value for the left (right) parotid gland and the left (right) submandibular gland was 4, with maximal sensitivity (75.6% and 77.2%, respectively) and specificity (91.6% and 92.2%, respectively). The pSS patients with positive SGUS results presented a longer disease duration, parotid enlargement, dental loss and higher levels of serological markers, such as anti-SSA, anti-SSB, positive RF, IgG and γ-globulin%.

**Conclusions:**

SGUS with the OMERACT scoring system yields high sensitivity and specificity, demonstrating high diagnostic feasibility for pSS. The SGUS may have implications for deciding disease severity and treatment efficacy.

## Background

Primary Sjögren’s syndrome (pSS) is a relatively common systemic autoimmune disease characterized by lymphocytic infiltration and exocrine gland destruction. Approximately 30 to 40% of patients also suffer from extraglandular manifestations of internal organs [1]. Higher risk of developing oncohemalogical disorders, such as non-Hodgkin lymphoma represents pSS pathognomonic hallmarks and become the major threats to patients with pSS [2, 3].

Currently, there have existed various imaging techniques in assessing the involvement of salivary glands in individuals with Sjögren’s syndrome (SS). However, considering that these techniques are mostly invasive and costly and can lead to irradiation, they are still not widely used in the assessment of patient outcomes in daily clinical practice. An increasing body of evidence including our previous study suggests that major salivary gland ultrasonography (SGUS) performs well in diagnosing SS, but the pathological features are defined differently across studies which apply distinct scoring systems [4–9]. Recently, to standardize SGUS, the Outcome Measures in Rheumatology Clinical Trials (OMERACT) working group has reached an international expert consensus on SGUS elementary lesion definitions and a novel four-grade semiquantitative scoring system (grade 0–3) for pSS [10]. The inter-rater and intra-rater reliability rates of the system were good and excellent, respectively [10, 11]. However, the diagnostic accuracy and feasibility for pSS and non-pSS sicca patients remain to be tested in clinical practice in large cohort studies.

Furthermore, some studies have shown that SGUS is associated with salivary gland swelling, cryoglobulinemic vasculitis, the production of rheumatoid factor (RF), immunoglobulin and germinal centre-like structures in salivary gland biopsies [5, 6, 12, 13]. Of note, previous studies have also provided evidence of SGUS changes after rituximab treatment and predicted the efficacy of xerostomia treatment for pSS [14–16]. Therefore, SGUS might be not only useful as a diagnostic tool but also suitable as a predictive and follow-up biomarker for disease activity and the risk of lymphoma. However, the benefit of SGUS for monitoring the natural history, predicting the outcomes and detecting lymphoma has yet to be investigated in daily clinical practice and larger cohort studies [17].

We aimed to evaluate the diagnostic value of the novel OMERACT scoring system for pSS in this large-scale multicentre study. The secondary aim was to investigate the associations between SGUS characteristics and systemic manifestations of pSS.

## Methods

### Study population

**Fig. 1:**
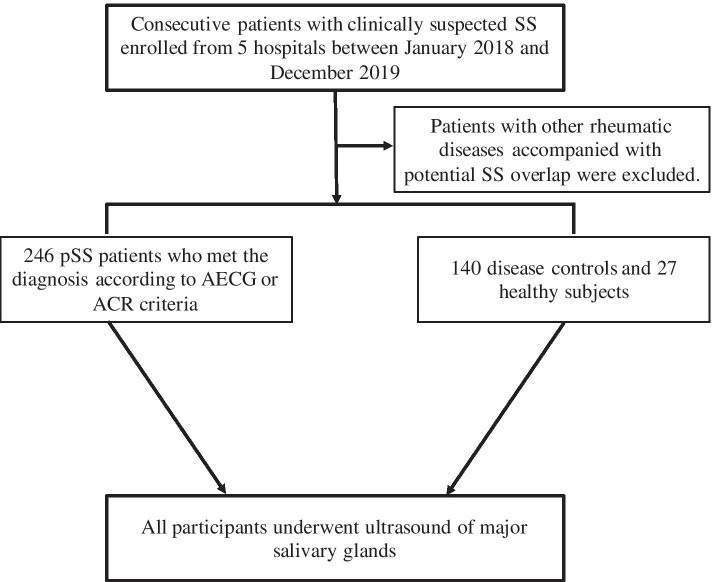
Flow chart of the study. AECG, American-European Consensus Group; ACR, American College of Rheumatology

This was a multicentre study with consecutive patients clinically suspected of having SS enrolled from 5 hospitals across different regions of China between January 2018 and December 2019 in daily clinical practice. All patients were aged ≥ 18 years and underwent a diagnostic workup for pSS according to the American-European Consensus Group (AECG) or American College of Rheumatology (ACR) criteria [18, 19]. The patients with other rheumatic diseases accompanied with potential SS overlap were excluded at the beginning of the study. Then, SGUS was conducted for 246 pSS patients, 140 patients with SS-related manifestations but without SS and 27 healthy subjects. A flow chart of the study design was presented in Fig. 1. The study was approved by the medical ethics committee of the Institute of Peking, all participants provided written consent, and the study was performed according to the guidelines of the Declaration of Helsinki.

### Clinical, serological and immunological assessment

All patients were subjected to diagnostic workups for pSS, which was performed without knowledge of SGUS results. The questionnaire-based evaluation included assessments of the following parameters: ocular symptoms; oral symptoms; ocular signs (Schirmer I test <5 mm in 5 min, ocular staining score >4 according to the van Bijsterveld scoring system); salivary gland involvement as determined using parotid sialography and salivary scintigraphy; positive histopathological examination results of biopsy specimens of the minor salivary gland (MSG) (defined as the number of mononuclear infiltrates containing ≥ 50 lymphocytes/4 mm^2^ of glandular tissue); serological tests, including those for anti-SSA, anti-SSB, antinuclear antibody (ANA), RF, immunoglobulin G (IgG) and γ-globulin% and other systemic manifestations such as cutaneous vasculitis, arthritis, leukopaenia, interstitial lung disease, renal tubular acidosis and peripheral/central nervous involvement.

### Salivary gland ultrasonography

All patients were examined by grey-scale and colour Doppler US by five well-trained examiners (S.S.Z., J.X.Z, Y.W., L.L., Y.J.C.) from different centres who were blinded to the clinical data. The US system was equipped with a high-resolution linear transducer (9–12 MHz). The bilateral parotid and submandibular glands were scanned. The novel four-grade semiquantitative scoring system (grade 0–3) developed for the OMERACT was employed to assess the echostructure of each gland [10]. The novel four-grade OMERACT semiquantitative scoring system (grade 0–3) was employed to assess the echostructure of each gland. The scores are defined as: grade 0, normal parenchyma; grade 1, mild inhomogeneity without anechoic or hypoechoic areas and hyperechogenic bands; grade 2, moderate inhomogeneity with focal anechoic or hypoechoic areas; and grade 3, severe inhomogeneity with diffuse anechoic or hypoechoic areas occupying the entire gland or a fibrous gland [10].

### Statistical analysis

All statistical analyses were performed using Statistical Package of Social Science (SPSS) software version 16.0. For statistical comparisons, the Mann-Whitney *U* test or chi-square test was used, as appropriate. Cohen’s kappa was used to analyse the concordance between the scores of bilateral salivary glands. Spearman’s test was used for correlation analysis. Receiver operating characteristic (ROC) curves were generated to determine the diagnostic performance of the scoring systems for pSS. On the ROC curves, the optimal cut-off point producing the maximal combination of sensitivity and specificity was located nearest the upper left corner of the curve. *P* values <0.05 were considered statistically significant.

## Results

### Characteristics of the study population

A total of 246 patients with pSS who fulfilled the AECG or ACR criteria were included (female/male ratio 239/7; age 53.16 (SD 12.13) years; symptom duration 4 (range 0.1–31) years). The control group comprised a total of 167 subjects: 113 non-SS patients with various rheumatic diseases (29 with undifferentiated connective tissue disease, 32 with connective tissue disease, 2 with mixed connective tissue disease, 28 with rheumatoid arthritis, 13 with systemic lupus erythematosus, 1 with sclerosis, 1 with systemic vasculitis, 7 with primary biliary cirrhosis), 27 with idiopathic Sicca syndrome and 27 healthy subjects (female/male ratio 156/11; age 51.21 (S.D. 13.72) years). The two groups were similar regarding age and gender (*P*=0.139 and 0.068, respectively). The characteristics of the pSS and non-pSS subjects are shown in Table 1.

**Table 1:** Characteristics of the study population

Characteristics	pSS patients (*n*=246)	Non-pSS subjects (*n*=167)	
		Healthy subjects (*n*=27)	Non-pSS patients (*n*=140)
Age, mean (S.D.), years	53.16 (12.13)	51.21 (13.72)	
Female/male	239/7	156/11	
Symptom duration Median (range), years	4 (0.1–31)*	0	2 (0.1–30)
Parotid enlargement	75 (30.5)*	0	3 (2.1)
Dental loss	95 (38.6)*	0	4 (2.9)
Schirmer <5 mm^a^	128/129*	0	17/33
Anti-SSA^a^	176/223*	0	44/130
Anti-SSB^a^	89/224*	0	15/125
MSG biopsy^a^	37/52*	0	0/1

Except where indicated otherwise, the values are presented as the mean (S.D.), median (range) or number (%). ^a^Values of objective tests given as rates of positive results (positive/total). The *p* values were determined using the Mann-Whitney test or chi-square test, as appropriate; *P < 0.05: statistically significant (regarding the difference between pSS patients and non-pSS subjects). *MSG* minor salivary gland

### Features of SGUS in pSS

**Fig. 2:**
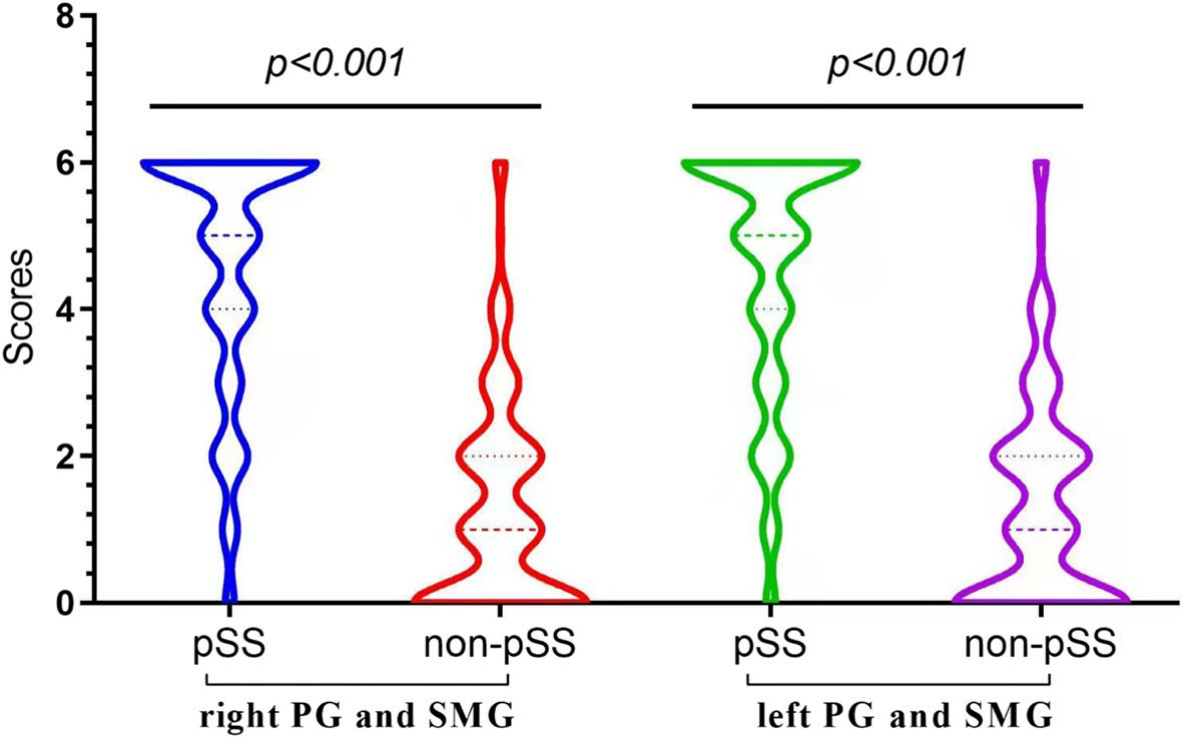
Features of SGUS scores. Distribution of the US scores for the right parotid and submandibular glands, left parotid and submandibular glands, and all four glands between pSS and non-pSS group. PG, parotid gland; SMG, submandibular gland

All patients and control group underwent SGUS scores which displayed no difference between bilateral parotid glands (*p* =0.798), followed by bilateral submandibular glands (*p*=0.842). Further, the kappa values indicated high concordance between the bilateral parotid glands for all participants (κ = 0.93, *p* <0.001), so did the bilateral submandibular glands (κ = 0.921, *p* <0.001), which indicated that the US scores of the same type of salivary glands were interchangeable (i.e., right vs left parotid gland; right vs left submandibular gland). The US scores of the right parotid and submandibular glands, the scores of the left parotid and submandibular glands and the sum of the scores of all four glands in the pSS group were significantly higher than those in the non-pSS group (*p* < 0.001, overall) (Fig. 2).

**Fig. 3:**
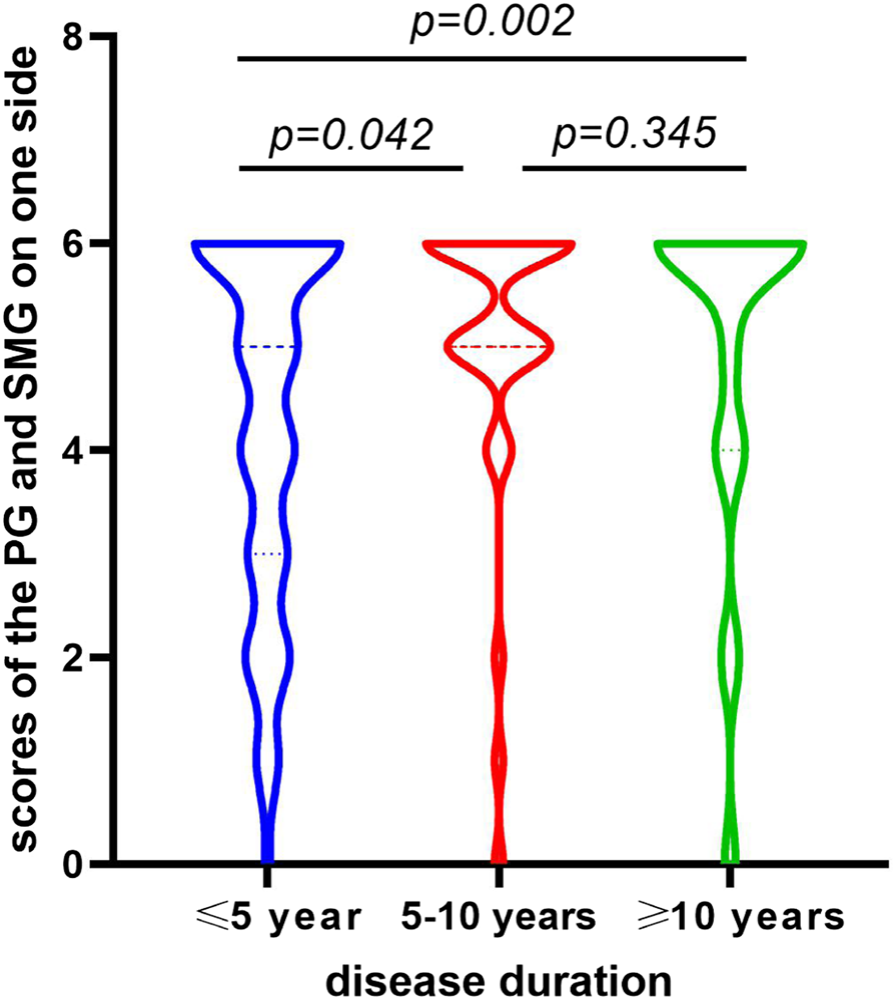
Association between the scores of the parotid and submandibular glands on one side with disease duration.PG, parotid gland; SMG, submandibular gland

Correlation analysis suggested a positive correlation between the scores of parotid and submandibular glands on one side and disease duration (Spearman *r*=0.208, *p* =0.001). Subsequently, to further assess the associations between the SGUS scores and disease duration, the pSS patients were further stratified according to symptom duration [symptom duration: first stage ≤ 5 years (*n*=151), second stage 5–10 years (*n*=35) and third stage ≥10 years (*n*=60)]. After the analysis based on one side among diverse groups, data showed a trend to have significant differences between the first and second stages and between the first and third stages (*p* =0.042, 0.002, respectively) but not between the second and third stages (*p* =0.345) (Fig. 3). Besides, when adjusting SGUS scores to age or gender, there also witnessed no statistical difference among all the subjects in our study.

### SGUS diagnostic accuracy for pSS

The diagnostic accuracy of the US scores of all four glands in diagnosing pSS was excellent (AUC=0.908, 95% CI 0.879, 0.938). Accuracy was comparable when the scores of only the right parotid and submandibular glands (AUC=0.910, 95% CI 0.881, 0.939) or the scores of only the left parotid and submandibular glands (AUC=0.904, 95% CI 0.875, 0.934) were included (*p* >0.05, overall) (Table 2). A US cut-off value of 7 provided maximal sensitivity (78%) and specificity (91.6%) for the scores of all four glands, while a US cut-off value of 4 showed maximal sensitivity (77.2% and 75.6%, respectively) and specificity (92.2% and 91.6%, respectively) for the left (right) parotid gland and the left (right) submandibular gland (Table 2), so these values were thought optimal.

**Table 2:** Diagnostic accuracy of the OMERACT scoring system for pSS

	Area under the curve (95% CI)	Cut-off value	SEN, %	SPE,%	PPV,%	NPV,%
Right PG	0.90 (0.869–0.930)	2	77.6	88.6	91.4	72.9
Right SMG	0.879 (0.845–0.912)	2	82.9	79.6	85.7	76
Left PG	0.893 (0.861–0.924)	2	76.0	89.8	91.7	71.8
Left SMG	0.874 (0.84–0.908)	2	83.3	77.8	84.7	76.0
Right PG + SMG^a^	0.910 (0.881–0.939)	4	77.2	92.2	93.6	73.3
Left PG + SMG^b^	0.904 (0.875–0.934)	4	75.6	91.6	93	71.8
Total four glands^c^	0.908 (0.879–0.938)	7	78	91.6	93.2	73.9

*P* <0.05 determined by the McNemar test was considered statistically significant. a vs b, *p* =0.508; b vs c, *p* =0.062, a vs c, *p* =0.625. *PG* parotid gland, *SMG* submandibular gland, *SEN* sensitivity, *SPE* specificity, *PPV* positive predictive value, *NPV* negative predictive value

### SGUS in clinical stratification

The cut-off value of the SGUS scores of the parotid and submandibular glands on one side allowed for the classification of patients with pSS: the positive SGUS group (SGUS score ≥4) and the negative SGUS group (SGUS score <4). The results suggested that 190 (77.2%) patients had positive SGUS findings, and 56 (22.8%) patients had negative SGUS findings. It was observed that the correlation between the positive and negative groups with respect to systemic manifestations including cutaneous vasculitis, interstitial lung disease and other extra-glandular manifestations, did not reach significance (*p* > 0.05, overall). However, one patient in the SGUS-positive group had cryoglobulinaemia and amyloidosis. Parotid swelling and dental loss were found to occur more frequently in patients with positive scores than in those with negative scores (*p* =0.008, 0.001, respectively) (Table 3).

**Table 3:** Characteristics of pSS patients with positive and negative SGUS results

	Negative (SGUS < 4)	Positive (SGUS ≥ 4)	*p*
*N* (%)	56 (22.8)	190 (77.2)	-
Age (S.D.), years	53.16 (12.99)	53.16 (11.91)	0.92
Female/male	54/2	185/5	0.71
Symptom duration, median (range), years	2 (0.1–27)	5 (0.1–31)	**< 0.001**
Parotid swelling	9 (16.1)	66 (34.7)	**0.008**
Dental loss	11 (19.6)	84 (44.2)	**0.001**
ANA ≥1:320	25 (48.1)	105 (61.8)	0.08
Anti-SSA positivity	33 (66.0)	143 (82.7)	**0.011**
Anti-SSB positivity	12 (24.0)	77 (44.3)	**0.01**
Positive RF	29 (58)	130 (81.8)	**0.001**
IgG, g/l	17.1 (6.6)	21.37 (8.34)	**0.001**
γ-globulin,%	21.74 (6.84)	25.62 (6.47)	**0.01**
C3, median (range), g/l	1.15 (0.88–1.49)	0.96 (0.64–1.66)	0.07
C4, median (range), g/l	0.18 (0.12–0.38)	0.17 (0.12–0.34)	0.951
Systemic complications			
Cutaneous vasculitis	6 (10.7)	25 (13.2)	0.628
Interstitial lung disease	14 (25)	50 (26.3)	0.844
Renal involvement	3 (5.4)	20 (10.5)	0.243
Nervous system involvement	3 (5.4)	10 (5.3)	0.978
Leukopaenia	14 (25)	59 (31.1)	0.384
Cryoglobulinaemia	0	1 (0.5)^a^	0.586

Except where indicated otherwise, the values are presented as the mean (S.D.), median (range) or number (%). The *p* values were determined using the Mann-Whitney test or chi-square test, as appropriate. ^a^One patient had cryoglobulinemia and amyloidosis. *PG* parotid gland, *SMG* submandibular gland, *ANA* antinuclear antibodies, *RF* rheumatoid factor, *IgG* immunoglobulin G, *C3* complement 3, *C4* complement 4

### Associations between SGUS and serological parameters

In light of the specific value of serological characteristics in diagnosing autoimmune diseases, the pSS patients with positive scores expressed a preference for autoantibodies to SSA and SSB and obviously higher levels of RF, IgG and γ-globulin% (*p* < 0.05, overall) (Table 3). However, the ANA results along with the level of complement in the positive SGUS group shared the similar information with the negative SGUS group (*p* > 0.05) (Table 3).

**Fig. 4:**
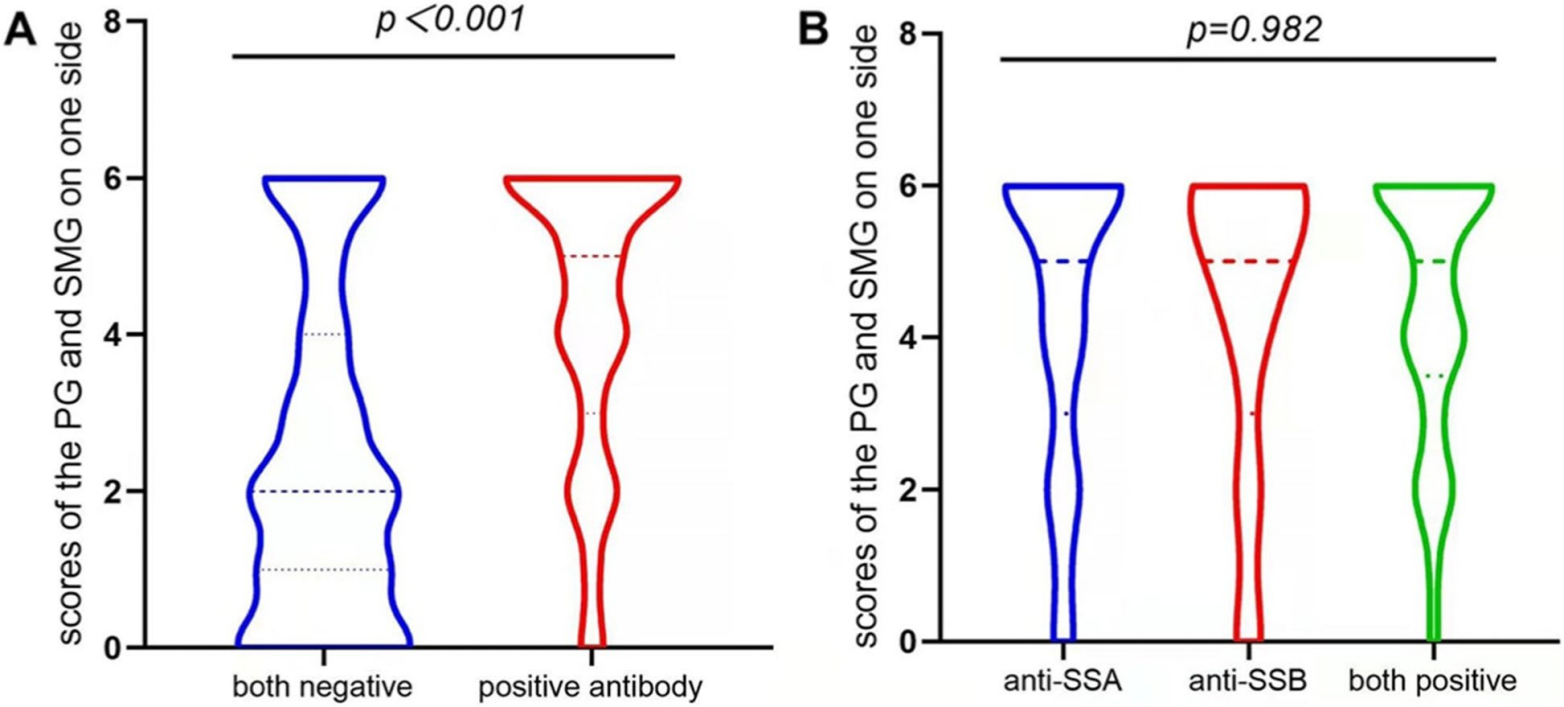
SGUS scores associated with the existence of any of antibodies. **A** patients with any positive antibodies compared to both negative. **B** No significant difference among groups including only anti-SSA, only anti-SSB antibodies and both positive

Given that the presence of autoantibodies is available for the classification of pSS and daily clinical practice, 239 individuals were evaluated with serum levels of anti-SSA or anti-SSB. Data showed that patients with any of antibodies were significantly involved in the higher SGUS scores (*p* <0.05) (Fig. 4A). Additionally, all the samples with positive antibodies were distributed into three groups including only anti-SSA, only anti-SSB antibodies and both positive. Our analysis revealed no significant difference among them (*p* =0.982) (Fig. 4B).

In our study, fifty-two patients in the pSS group underwent labial gland biopsies. Among the 52 patients, SGUS score ≥4 of the parotid and submandibular glands on one side and positive biopsy findings were recorded in 38/52 (73.1%) and 37/52 (71.2%) patients, respectively (*p* > 0.05). The absolute agreement between SGUS scores and labial gland biopsy was 67.3% (35/52).

## Discussion

There is a growing number of evidence suggesting that SGUS has become an integral component in a thorough grasp of typical structural abnormalities of major salivary glands in pSS [4–9]. SGUS has the potential to be used for pSS classification and as a follow-up tool, but for these purposes, additional research, standardized procedures and larger cohort studies are needed [17]. As for the multitude of SGUS abnormalities that occur in individuals with pSS, it is challenging to reach a consensus on the definition and scoring method most appropriate for the most common SGUS abnormalities. By offering an updated classification system, a novel four-grade semiquantitative scoring system, the OMERACT group undeniably advanced the diagnosis of pSS [10]. Its intra-rater reliability was excellent and inter-rater reliability was good [10, 11]. In the next step, the new scoring system needed to be tested in a large number of patients with pSS and non-pSS sicca symptoms.

In this multicentre large-scale study, we confirmed the high diagnostic performance of SGUS to discriminate pSS and symptomatic sicca control patients with the new OMERACT scoring system. The diagnostic accuracy of the US scores of all four glands to diagnose pSS was outstanding and embraced comparable results while only depending on the scores of the parotid and submandibular glands on one side. There was an apparently high correlation between the same type of salivary glands. Our previous study showed that the scores for the submandibular glands on both sides were significantly higher than those for the parotid gland, indicating that there was a weak association between the parotid and submandibular glands [5]. Similar results were reported by Mossel E et al. [20]. An optimal US cut-off value of 7 provided maximal sensitivity (78%) and specificity (91.6%) for the scores of all four glands, while an optimal US cut-off value of 4 showed comparable sensitivity (77.2% and 75.6%, respectively) and specificity (92.2% and 91.6%, respectively) for the left (right) parotid gland and the left (right) submandibular gland. Fana V et al. reported that the best ultrasound cut-off value for diagnosing pSS was ≥1 gland with a score ≥2 (sensitivity=72%, specificity=91%), in which the diagnostic accuracy was similar to our results [21]. Thus, this new scoring system for the left (right) parotid gland and the left (right) submandibular gland showed high diagnostic performance and greatly increased the feasibility of SGUS as a routine procedure in pSS. Given that some patients have unilateral complaints or a high risk of lymphoma, such as parotid enlargement, a global view of all four glands should be taken into consideration, but only one side might be feasible to be scored if there was no particular disease to differentiate.

In addition to the diagnostic value of SGUS, previous studies have shown a correlation between SGUS characteristics and the clinical and serological features of pSS, indicating that SGUS may hold promise for assessing disease activity and treatment efficacy [5, 6, 22]. However, investigations of its usefulness for monitoring the natural history, predicting the outcomes and detecting lymphoma are extremely challenging to perform. In this large-scale multicentre study, we found that the SGUS scores were related to disease duration, parotid swelling, dental loss and the levels of anti-SSA, anti-SSB, RF, IgG and γ-globulin%. Cryoglobulinaemia (a well-known risk factor for progression to B lymphoma) and amyloidosis were present in one patient in the SGUS ≥4 group, and no cases of cryoglobulinamia were found in patients with negative SGUS findings. Thus, SGUS was deemed to be related to the usual risk factors for lymphoma of parotid swelling and B cell hyperactivity, regarding polyclonal or monoclonal hyperglobulinaemia and the production of RF. Consistent with the findings of previous studies, Theander and Mandl showed that SGUS enabled patients with systemic complications, high disease activity and lymphoma development to be identified [6]. In addition, Guillaume et al focused on the association between SGUS characteristics and systemic complications and found that SGUS exhibited great benefit in supervising pSS patients with cryoglobulinemic vasculitis or lymphoma [12]. This provides the evidence that SGUS features might be evaluated as a follow-up strategy and may be predictive markers for lymphoma. No cases of lymphoma were found in any of the pSS patients in the present study, probably since the follow-up period was not sufficiently long. Therefore, future longitudinal studies for SGUS should recruit a larger cohort and monitor the risk of lymphoma over time during the natural course of the disease to shed more light on this challenging issue.

Salivary gland abnormalities due to pSS are believed to progress over time, resulting in changes in SGUS features. However, SGUS scores have not been found to be affected by disease duration. Pierre et al. observed that SGUS abnormalities were already present at diagnosis while did not change substantially over the first few years in patients with pSS [23]. Kyung-Ann et al. reported no significant differences between pSS patients with a disease duration ≤5 years and those with a disease duration >5 years with respect to the SGUS scores [22]. These findings suggest that pSS may remain asymptomatic for many years, exert a considerable burden on interpreting the time of disease onset. In the present large-scale study, statistically significant differences were observed between disease durations ≤5 years and 5–10 years and between ≤ 5 and ≥ 10 years, but not between 5–10 and ≥10 years. SGUS abnormalities of hypoechoic areas seem detectable at the early stages of the disease, while diffuse inhomogeneity with anechoic/hypoechoic areas or fibrosis is found in end-stage pSS. According to the novel OMERACT scoring system, SGUS abnormalities with diffuse anechoic/hypoechoic areas or salivary gland fibrosis were defined as grade 3 abnormalities [10]. Therefore, the new scoring system enjoys an effective position on managing well-established diseases at the time of diagnosis whereas restricted in discriminating pSS cases in the middle and late stages.

Importantly, our study did have certain limitations that firstly, some of the SGUS images were scored retrospectively because the novel OMERACT scoring system was published online in April 2019. Moreover, we did not follow up patients to monitor the development of lymphoma after the ultrasound examination. Future longitudinal large-scale studies for SGUS are warranted to determine the risk of lymphoma throughout the natural course of the disease.

## Conclusions

In conclusion, this large-scale multicentre study showed that SGUS using the novel OMERACT scoring system demonstrated high sensitivity and specificity for the diagnosis of pSS with good feasibility. This method is also of great value for monitoring disease characteristics. Therefore, SGUS may serve as a widely-adopted approach in the diagnosis, global follow-up and management of pSS.

## Data Availability

All relevant generated data and material are included in the manuscript.

## Abbreviations

pSS: Primary Sjögren’s syndrome
SGUS: Salivary gland ultrasonography
OMERACT: Outcome Measures in Rheumatology Clinical Trials
RF: Rheumatoid factor
AECG: American-European Consensus Group
ACR: American College of Rheumatology
MSG: Minor salivary gland
ANA: Antinuclear antibody
IgG: Immunoglobulin G
SPSS: Statistical Package of Social Science
ROC: Receiver operating characteristic
PG: Parotid gland
SMG: Submandibular gland
SEN: Sensitivity
SPE: Specificity
PPV: Positive predictive value
NPV: Negative predictive value
C3: complement 3
C4: complement 4
